# Dietary High-Fat Promotes Cognitive Impairment by Suppressing Mitophagy

**DOI:** 10.1155/2023/4822767

**Published:** 2023-01-21

**Authors:** Jie Wen, Yangyang Wang, Chuanling Wang, Minghao Yuan, Fei Chen, Qian Zou, Zhiyou Cai, Bin Zhao

**Affiliations:** ^1^Department and Institute of Neurology, Guangdong Medical University, Zhanjiang, Guangdong 524001, China; ^2^Guangdong Key Laboratory of Aging-Related Cardiac and Cerebral Diseases, Zhanjiang, Guangdong 524001, China; ^3^Chongqing Key Laboratory of Neurodegenerative Diseases, Yuzhong District, Chongqing 400013, China; ^4^Department of Neurology, Chongqing General Hospital, Yuzhong District, Chongqing 400013, China; ^5^Chongqing Medical University, Yuzhong District, Chongqing 400016, China

## Abstract

Dietary habits contribute to the characteristics of Alzheimer's disease (AD) and cognitive impairment, which are partly induced by the accumulation of hyperphosphorylated Tau, a microtubule-associated protein. In mice, a fat-rich diet facilitates cognitive dysfunction. However, the mechanism by which dietary fat damages the brain remains unclear. In this study, 13-month-old C57BL/6 mice were fed a normal or high-fat diet (HFD) for 6 months. Neuro-2a cells were incubated with the normal medium or palmitic acid (200 *μ*M). Spatial memory was assessed utilizing a behavioral test. Further, western blotting and immunofluorescence techniques were used to determine the levels of mitophagy-related proteins. The synaptic morphology and phosphorylation of Tau proteins were also evaluated. Administration of HFD decreased the expression of synaptophysin and brain-derived neurotrophic factor expression, leading to significant damage to neurons. Tau protein hyperphosphorylation was detected at different loci both *in vivo* and *in vitro*. Significantly impaired learning and memory abilities, accompanied by impaired mitophagy-related processes, were observed in mice fed with HFD as compared to mice fed with normal food. In conclusion, high fatty-acid intake hinders mitophagy and upregulates Tau protein phosphorylation, including age-related synaptic dysfunction, which leads to cognitive decline.

## 1. Introduction

High-fat foods, which are characteristic of Western diets, are associated with metabolic disorders, including obesity and diabetes, central nervous system inflammation, and impaired cognitive performance [1, 2]. The increased consumption of saturated fats is closely related to the rising epidemic of metabolic diseases. In a rat model, a high-fat diet (HFD) was found to impair hippocampal and frontal cortical performance in various tasks, which was associated with levels of saturated fatty acids [3]. In addition, epidemiological findings suggest that the intake of polyunsaturated and short-chain fatty acids has beneficial effects on cognitive performance; in contrast, a high intake of saturated fats and cholesterol is associated with cognitive decline [4]. However, the mechanisms by which fatty acids alter cognitive function remain only partially understood, with Tau protein being of major interest in this regard. The hyperphosphorylation of Tau protein leads to its physiological dysfunction and accumulation. This forms neurofibrillary tangles, which has neurotoxic effects and is considered an essential pathological feature in Alzheimer's disease (AD). Researches have revealed that HFDs accelerate cognitive impairment in AD mice [5–7] and senescence-accelerated prone 8 mice, which show elevated amyloid-*β* (A*β*) accumulation and excessive Tau protein phosphorylation [8].

Various traits of aging in the brain are closely related to mitochondrial dysfunction. The mitochondria, which serve as cellular energy power plants, provide adenosine triphosphate (ATP) to fuel various activities that are necessary for neuronal function and survival. ATP depletion at synapses drives action and synaptic potentials and powers the transport, repopulation, and recycling of synaptic vesicles, thereby maintaining neurotransmission. The formation of axon terminals and synapses is essential for establishing and modifying neuronal connections, because the proper functioning of the central nervous system depends on the establishment of precise neuronal connections [9]. Aged and compromised mitochondria function poorly and release harmful reactive oxygen species (ROS) that can affect mitochondria-mediated synaptic activity and neuronal health. High concentrations of saturated fatty acids in neuronal mitochondria have been reported to undermine several processes of ATP formation, owing to electron leakage and mitochondrial ROS production. These processes enhance oxidative stress and mitochondrial lesions, which can cause cellular hypoxia and provoke apoptosis [10]. Further, high saturated fatty acid levels are associated with insulin resistance in peripheral tissues. Although not insulin-dependent, hippocampal and cortical neurons respond to insulin, suggesting its vital role in regulating metabolism, growth, synapses, and overall cell survival and function [11]. Systemic and neuronal insulin resistance have been reported in the hippocampus and hypothalamus after administering HFD to rodents [12].

Microtubule integrity is crucial for dynamic mitochondrial movement within cells. In neurons, the mitochondria primarily move from the soma to the axon to supply energy to these cells [13]. The mitochondria provide energy to the dynamic microtubules and maintain their integrity; thus, they support cellular organization, which is essential for behaviors such as cellular proliferation and migration [14]. During cell growth and migration, the mitochondria produce bioenergy to sustain the assembly of microtubule-associated proteins for microtubule polymerization [15]. Mitochondrial abnormalities, including morphological features, have been identified in both the early stages of AD and in experimental AD tauopathy models [16, 17]. Additionally, mitophagy represses Tau pathology, and phosphorylated Tau has been shown to damage the mitochondria by impairing the complex I activity of the mitochondrial respiratory chain and altering mitochondrial dynamics and transport [18–20]. Consequently, the interaction of mitochondrial dysfunction with Tau pathology may lead to a vicious cycle that exacerbates the development of neurodegenerative diseases. In addition, PINK1 is pronounced in mitochondrial disorders during early tauopathy. This precedes synaptic dysfunction and microtubule-associated Tau protein/Tau dysregulation and manifests as depolarized mitochondrial membrane potential (*Δψ*m) and elevated oxidative stress, which is reflective of diminished energy metabolism [9]. However, the relationship between mitophagy, Tau hyperphosphorylation, and synaptic dysfunction in HFDs remains elusive.

Based on previous findings, we hypothesized that excessive and toxic lipid accumulation might lead to mitochondrial dysfunction by inhibiting mitophagy, which could lead to Tau protein hyperphosphorylation and synaptic dysfunction, and eventually result in cognitive impairment. In this study, we aimed to determine the effect of high lipid consumption on mitophagy, Tau protein hyperphosphorylation, and synapses in order to investigate the potential link between mitophagy and high-fat-induced synaptic dysfunction or Tau protein hyperphosphorylation, which could have implications for future studies.

## 2. Material and Methods

### 2.1. Animals

Male C57BL/6 mice (13 months, weighing 24–34 g) were provided by Chengdu Dossy Experimental Animals Co., Ltd. (Chengdu, China) and housed under a 12 h light/dark cycle in a suitable temperature environment (25 ± 2°C) at the Laboratory Animal Center of the Chongqing Key Laboratory of Neurodegenerative Diseases (Chongqing, China). Mice were randomly grouped and housed with other mice in cages (2–4 mice per cage). All animal experiments were approved by the Research Ethics Committee of Chongqing Medical University.

### 2.2. High-Fat Diet

Male mice had ad libitum access to tap water and were given normal chow (normal diet, ND) (18% protein, 78% carbohydrate, and 4% fat, #1025; HFK Bioscience, Beijing, China) or fat-rich chow (high-fat diet, HFD) (20% protein, 20% carbohydrate, and 60% fat, #D12492; ReadyDietech Biotechnology, Shenzhen, China) for 24 weeks.

### 2.3. Cell Culture and Treatment

Neuro-2a (N2a; mouse neuroblastoma cells) was provided by Procell Life Science&Technology Co., Ltd. N2a cells were cultured in Dulbecco's Modified Eagle Medium Nutrient Mixture F-12 (DMEM/F12, Gibco, Carlsbad, CA, USA) and supplemented with 10% fetal bovine serum (FBS, Invigentech, Irvine, CA, USA) and antibiotics (100 U/ml penicillin and 100 *μ*g/ml streptomycin). The culture was incubated in a humidified atmosphere with 5% CO_2_ at 37°C. After 24 h of differentiation, the cells were disposed of normal medium, palmitic acid (200 *μ*M for 24 h) [21] (Kunchuang Biotechnology, Xi'an, China) and palmitic acid + Kaem (kaemferol, 50 *μ*M for 24 h) (HY-14590, MCE).

### 2.4. *β*-Galactosidase Staining

The *β*-galactosidase staining was undertaken by utilizing a senescence-related *β*-galactosidase staining kit (Beyotime Biotechnology, Shanghai, China). Cells were fixed with *β*-galactosidase staining fixative for 15 min. The cells were then incubated with a working solution containing 0.05 mg/ml 5-bromo-4-chloro-3-indolyl-*β*-d-galactopyrano-side overnight at 37°C. Finally, they were observed under a fluorescence microscope.

### 2.5. *ΔΨm* In N2a Cell

For the JC-1 assay (C2003S, Beyotime Biotechnology, Shanghai, China) to assess mitochondrial membrane potential (*ΔΨ*m) in N2a cells, the cells were first inoculated into 6-well plates. Next, PA and Kaem were added to the corresponding experimental group after the cells adhered to the plates. After 24 h of growth, 1 ml of cell culture fluid and JC-1 staining working solution was added. They were incubated at 37°C for 20 min. Then, they were washed twice and observed using fluorescence microscopy. The excitation and emission wavelengths were 514 nm and 529 nm, respectively, and the monomeric form of JC-1 was detected. The aggregation of JC-1 was detected with 585 nm and 590 nm. The ratio of the aggregated JC-1 to the monomeric JC-1 was *ΔΨ*m.

### 2.6. Morris Water Maze

The Morris water maze (MWM) was aimed to measure spatial learning and memory in mice after being fed with an HFD for 6 months. An escape platform with a diameter of 8 cm in the northeast quadrant of the circular tank (diameter 1.2 m, depth 0.4 m, and temperature 25 ± 1°C) was provided. Acclimated mice were trained 4 times daily for 6 days as an acquisition test. At the start of each experiment, each mouse was placed in a different quadrant and allowed to swim freely for 60 s. After the mice climbed onto the platform, they were allowed to stay there for 5 s (if a mouse did not find the platform within 60 s, an additional 30 s was given to guide the mouse to the platform). At the end of each training round, the mice were immediately taken out and dried. ANY-maze video tracking software (Stoelting Co., Wood Dale, USA) was used to record the escape latency and swimming trajectory. On the seventh day, the platform was removed from the pool, and the mice were placed in the opposite quadrant. The time, distance covered in the target quadrant, and the number of platform crossings were recorded for a 60 s period using a video system and ANY-maze software [22].

### 2.7. Western Blotting

Protein extracts from the mice were removed from the midbrain, diencephalon, cerebellum, and brainstem, leaving the cortex and hippocampus. Radioimmunoprecipitation assay lysis buffer containing the protease inhibitor phenylmethylsulfonyl fluoride was used to lyse the cell lysates, cortex, and hippocampus from the mice. The extracted total proteins were detected using a BCA protein analysis kit (Beyotime Biotechnology, Shanghai, China). The equivalent proteins were separated on gels of corresponding concentrations and then transferred to polyvinylidene fluoride membranes (Bio-Rad, Hercules, CA, USA). Blocked cell membranes were incubated with anti-PINK1 (#29297, Signalway Antibody LLC, Greenbelt, USA), Parkin (#4211, Cell Signaling Technology, Danvers, USA), LC3A/B (#12741, Cell Signaling Technology), SQSTM1/p62 (#23214, Cell Signaling Technology), beclin1 (#3495, Cell Signaling Technology), brain-derived neurotrophic factor (BDNF, 28205-1-AP, Proteintech Group, Inc., Chicago, USA), postsynaptic density protein 95 (PSD95, 20665-1-AP, Proteintech Group, Inc.), synaptophysin (SYN, 17785-1-AP, Proteintech Group, Inc.), Tau46 (#4019, Cell Signaling Technology), Phospho-Tau (Thr205) (#49561, Cell Signaling Technology), Phospho-Tau (Thr181) (#12885, Cell Signaling Technology), Phospho-Tau (Thr231) (#71429, Cell Signaling Technology), Phospho-Tau (Ser404) (#20194, Cell Signaling Technology), glyceraldehyde-3-phosphate dehydrogenase (AF0006, Beyotime Biotechnology), and *β*-actin (AF5003, Beyotime Biotechnology) at 4°C overnight. Next, the membranes were incubated with secondary antibodies for 1 h at room temperature. Protein bands were observed using Immobilon Western chemiluminescent horseradish peroxidase substrate (Millipore Corporation, Billerica, MA, USA) and the Tanon-5200 multiplex chemiluminescent analysis system (Tanon, Shanghai, China) [22].

### 2.8. Immunofluorescence Staining

Whole mice brains were removed and fixed with 4% paraformaldehyde for 48 h. Specimens were cut into 2 *μ*m paraffin sections with a rotating slicer (HM 340E, Thermo Scientific) in the sagittal plane along the long axis. Next, they were blocked with 5% goat serum albumin and incubated with primary antibodies PINK1 (#29297, Signalway Antibody LLC), Parkin (14060-1-AP, Proteintech Group, Inc.), and LC3A/B (#12741, Cell Signaling Technology) at 4°C overnight. After incubation with the secondary antibody, i.e., Alexa Fluor 488-coupled antibody (ZSGB-BIO, Beijing, China) for 1 h in the dark, staining was performed using DAPI (Beyotime Biotechnology) at 37°C for 8 min. A NEXCOPE microscope (NE900, Ningbo Yongxin Optics Ltd., Ningbo, China) was used for detection [22].

### 2.9. Transmission Electron Microscopy

The hippocampus was prefixed with 3% glutaraldehyde and then postfixed in 1% osmium tetroxide, followed by dehydration in series acetone (50%, 70%, 80%, 90%, and 95%), and subsequently, 100% acetone was used three times. Samples were infiltrated in Epox 812 for longer embedding. The approximately 60 to 90 nm ultrathin sections were prepared using an ultrathin slicer. Sections were observed with a JEM-1400-FLASH Transmission Electron Microscope (JEOL Ltd., Tokyo, Japan).

### 2.10. Statistical Analyses

GraphPad Prism (version 8.3.0; GraphPad Software Inc., San Diego, USA) was used to analyze the experimental data. Based on the normality test results, a two-sample *t*-test was applied to compare the differences in the measurement data between the two groups. A one-way analysis of variance (ANOVA) with Tukey's follow-up test was used for multiple comparisons. Data were expressed as mean ± standard deviation (SD), and differences were considered statistically significant at ^∗^*p* < 0.05, ^∗∗^*p* < 0.01, ^∗∗∗^*p* < 0.001, and ^∗∗∗∗^*p* < 0.0001.

## 3. Results

### 3.1. Hair Thinning and Weight Gain in HFD-Fed Mice

**Figure 1:**
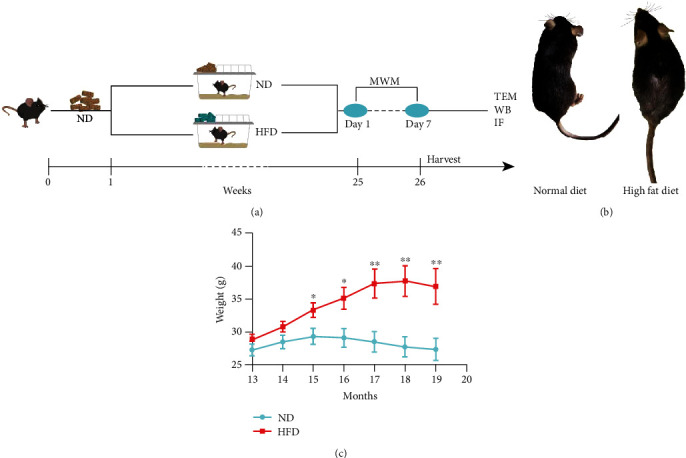
Hair and body weight characteristics of C57BL/6 mice that received ND or HFD. (a) Mice (13-month-old C57BL/6) were fed HFD or ND for 6 months. (b) The hair of mice in the HFD group was slightly sparse, dull, and without luster compared with the ND group. (c) The two groups differed in terms of body weight. *n* = 10/group, two-sample *t*-test. Data are shown as the mean ± SD; ^∗^*p* < 0.05, ^∗∗^*p* < 0.01. Abbreviations: HFD: high-fat diet; ND: normal diet; TEM: transmission electron microscopy; WB: western blotting; IF: immunofluorescence.

The mice were fed an HFD for 24 weeks (Figure 1(a)) to investigate the effects of dietary fat. Compared with their control counterparts, mice on HFD had yellowish, sparse, and dull hair (Figure 1(b)). Starting at baseline (13 months of age), both groups of mice that were fed HFD and standard chow fed gained a significant amount of weight over time; however, the mice that received HFD exhibited a more comprehensive range of body weight changes (Figure 1(c)). These data demonstrate that receiving HFD for a long time causes phenotypic changes in mice.

### 3.2. HFD Promotes Cognitive Impairment

**Figure 2:**
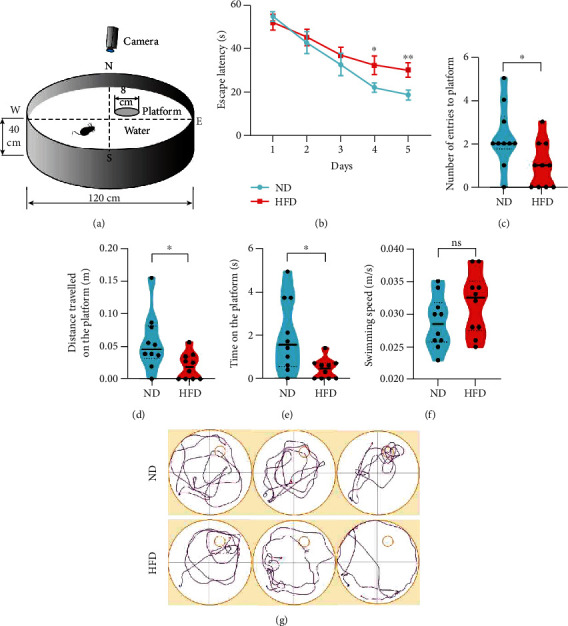
HFD aggravates spatial learning and memory abilities. (a) Schematic diagram and behavioral tests in aged C57BL/6 mice. C57BL/6 mice (13-month-old) were divided into two groups: the ND group was fed a normal diet, and the HFD group was fed a fat-rich diet for 6 months. HFD led to significant changes in the average escape latency (time to find the platform) (b), number of entries to the platform (c), time on the platform (d), and distance traveled on the platform (e), as compared to ND. There was no difference in the swimming speed between ND and HFD mice (f). Representative illustrations show the trajectories of mice in the final probe test in each experimental group (g). *n* = 10/group, two-sample *t*-test. Data are shown as the mean ± SD, ^∗^*p* < 0.05, ^∗∗^*p* < 0.01. Abbreviations: HFD: high-fat diet; ND: normal diet.

It has previously been shown that obesity induced by HFD impairs neurogenesis [23], potentially leading to neurodegeneration. Therefore, we performed the MWM to explore the potential effects of dietary fat intake on spatial learning and memory (Figure 2(a)). Both groups showed a time-dependent decrease in escape latency after consecutive training sessions; however, the mice in the HFD group spent more time searching for the hidden platform than the mice in the ND group (days 4 and 5; Figure 2(b)). In the probe session, the HFD group had a significantly shorter exploration time on the platform, shorter distance traveled on the platform, and fewer crossings of the virtual platform location (Figures 2(c)–2(e)) than the ND group. There was no difference in the swimming speed between the two diet groups (Figure 2(f)), which ruled out the possibility that the differences were a function of the swimming ability of the mice. The trajectories of the mice during the space exploration task in the ND and HFD groups are shown in Figure 2(g). Together, these findings indicate that feeding HFD to aged mice can exacerbate the progression of cognitive impairment and diminish object recognition and spatial memory abilities.

### 3.3. Enhanced Mitophagy Ameliorates PA-Induced Cellular Senescence

**Figure 3:**
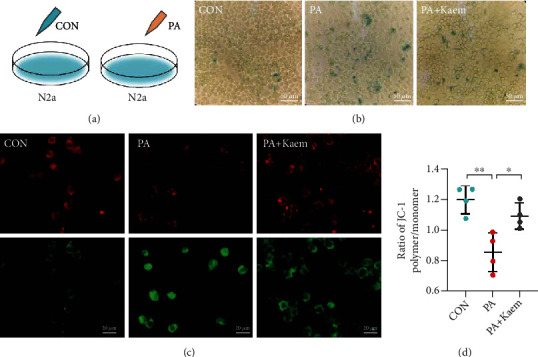
Enhanced mitophagy ameliorates PA-induced cellular senescence. (a) N2a cell treatment protocol. (b) Compared with the control group and PA + Kaem group, PA-exposed cells showed more senescent cell morphology and stained positive for SA-*β*-Gal. Scale bar = 50 *μ*m. (c) Red fluorescence represents the aggregated forms of JC-1 mitochondria, suggesting an intact mitochondrial membrane potential. The green fluorescence represents the monomeric form of JC-1, suggesting a lowered membrane potential. (d) The relative proportion of cells with red and green fluorescence was used to measure mitochondrial depolarization (*n* = 4/group, one-way ANOVA). Scale bar = 20 *μ*m. Data are shown as mean ± SD; ^∗^*p* < 0.05, ^∗∗^*p* < 0.01. Abbreviations: N2a: Neuro-2a; Kaem: kaemferol; CON: control; PA: palmitic acid.

Many hallmarks of aging are associated with mitochondrial dysfunction. Dysfunctional mitochondria are degraded through mitophagy, which contributes to mitochondrial turnover and to the preservation of mitochondrial stability. Therefore, we used a *β*-galactosidase assay to detect senescent cells. The A*β*-galactosidase staining kit uses X-Gal as a substrate and produces a dark blue product that is catalyzed by senescence-specific *β*-galactosidase. Senescent cells that turn blue can be easily visualized under an optical microscope. As shown in Figure 3(b), N2a cells treated with PA showed increased *β*-galactosidase activity and an increased volume of senescent cells as compared to the control group, suggesting that the high-fat diet induced cell senescence. Kaemferol (Kaem) has been shown to induce PINK1/Parkin-mediated mitophagy [24]. We further investigated whether Kaem treatment could prevent cell senescence induced by high fat, and the results showed that the senescence state of cells was alleviated after Kaem administration. This suggests that senescence induced by high fat may be related to mitophagy.

Next, we examined whether high fat damages mitochondria. The enhanced mitochondrial membrane potential assay kit with JC-1 uses it as a fluorescent probe for rapid and sensitive detection of cellular mitochondrial membrane potential changes. Normally, JC-1 is present as a J-aggregate and exhibits red fluorescence. However, JC-1 turns into a monomer in cells with mitochondrial damage and subsequently exhibits green fluorescence. Figures 3(c) and 3(d) illustrate that the ratio of cells exhibiting green fluorescence increased after PA treatment, indicating mitochondrial depolarization and suggesting mitochondrial dysfunction. Similarly, when PA was exposed to Kaem, the ratio of cells exhibiting red fluorescence increased, indicating that mitochondrial damage was improved.

### 3.4. Mitophagy Is Inhibited in the HFD/PA Group

**Figure 4:**
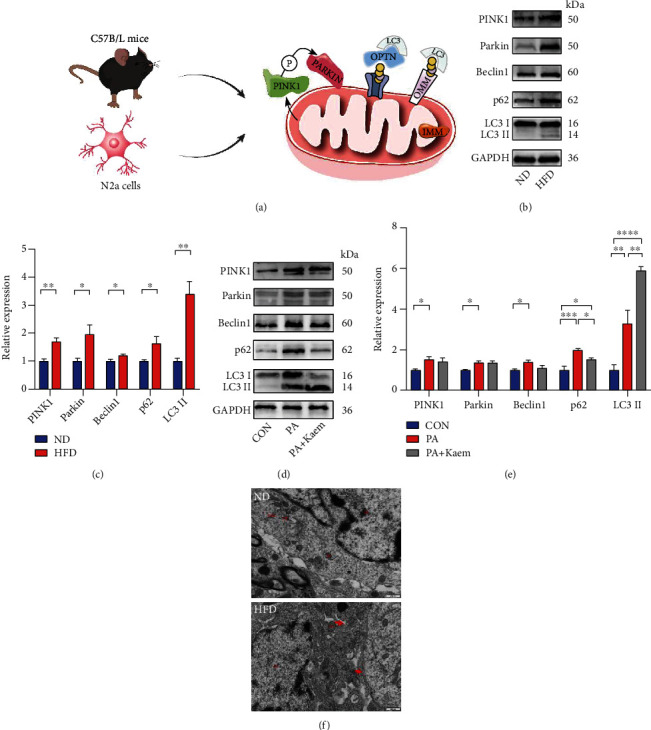
Expression of proteins related to mitophagy and mitochondrial morphology. (a) Schematic diagram of the mitophagy. (b, c) The western blotting indicated that HFD reduced the expression of PINK1, Parkin, Beclin1, and LC3II and increased the expression of p62 in the brains of aged C57BL/6 mice (*n* = 4/group, two-sample *t*-test). (d, e) The expression of proteins related to mitophagy in N2a cells is treated with PA medium (200 *μ*M). This expression profile was in line with the *in vivo* results (*n* = 4/group, one-way ANOVA). Data are shown as mean ± SD, ^∗^*p* < 0.05, ^∗∗^*p* < 0.01, ^∗∗∗^*p* < 0.001, and ^∗∗∗∗^*p* < 0.0001. (f) The mitochondrial structure in the hippocampal cytoplasm in the ND group was intact and clear. However, some neurons in the HFD group were necrotic, and most mitochondria in the cytoplasm appeared mildly swollen. N: nucleus; Mi: mitochondria. Scale bar = 500 nm. Abbreviations: ND: normal diet; HFD: high-fat diet; CON: control; PA: palmitic acid; GAPDH: glyceraldehyde-3-phosphate dehydrogenase.

We then used western blotting to confirm the protein expression levels of PINK1/Parkin mitophagy in the brains of aged C57BL/6 mice as well as cell models (Figure 4(a)). As shown in Figures 4(b) and 4(c), the levels of PINK1 and Parkin were enhanced in the HFD group compared to that in the ND group, indicating mitochondrial damage. In addition, both Beclin-1 and LC3II/LC3I were significantly increased. However, SQSTM1/P62 was enhanced in HFD mice. Autophagy deficiency causes the accumulation of P62 [25], which is an autophagy substrate and has been widely used as a predictor of autophagy flux [26]. Under certain stress conditions, the increase in autophagosome synthesis may be related to a decrease in lysosome activity. During lysosome deficiency, autophagosome production/accumulation directly induces cytotoxicity, since all lysosome-related pathways are dysfunctional and autophagosomes cannot be cleared and recycled when lysosomes are defective [27]. Our *in vitro* model of an HFD using N2a cells was consistent with the *in vivo* results (Figures 4(d) and 4(e)). Overall, we found that feeding HFD to the mice resulted in disruption of the normal flux of autophagy. In addition, we examined the levels of mitophagy-associated proteins after Kaem administration, which is a robust mitophagy inducer both *in vitro* and *in vivo* [24]. We observed that Kaem reduced the accumulated levels of P62 and contributed to the conversion of MAP1LC3-I to MAP1LC3-II (Figures 4(d) and 4(e)), which led to effective amelioration of their destructive effect *in vitro*. This suggests that Kaem could increase autophagosomes and enhance autophagy flux in PA-treated N2a cells.

Using transmission electron microscopy (TEM), we observed that neuronal cells in the ND group had irregular polygonal nuclei, uniform chromatin distribution, predominantly euchromatin, prominent nucleoli, and clear and complete nuclear membranes. Also, clear and complete mitochondrial structures were seen in the cytoplasm. In contrast, in the HFD group, some neuronal cells were apoptotic (nuclei were crinkled, chromatin was aggregated, and the cytoplasmic electron density was increased); moreover, some neuronal cells were necrotic (discontinuous cell membranes), and most mitochondria in the cytoplasm appeared mildly swollen (Figure 4(f)).

**Figure 5:**
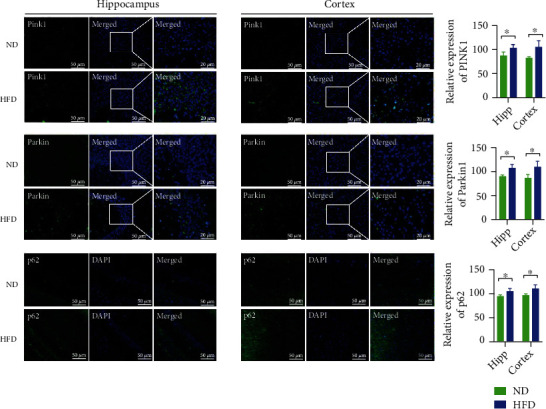
Immunofluorescence staining of proteins related to mitophagy. The expression of mitophagy-related proteins in the CA3 or CA1 areas of the hippocampus and in the cortex was observed by immunofluorescence (*n* = 3/group, two-sample *t*-test). All data are shown as mean ± SD; ^∗^*p* < 0.05. Scale bar = 50 *μ*m and 20 *μ*m. Abbreviations: ND: normal diet; HFD: high-fat diet; Hipp: hippocampus.

Additionally, immunofluorescence was performed to analyze the expression of PINK1, Parkin, and p62 in the two groups. The high-fat treatment group showed an increased fluorescence intensity of PINK1, Parkin, and p62 (Figure 5) compared with the control group, as per the results obtained from western blotting. These experimental data suggest that HFD induces neuronal and mitochondrial damage and inhibits mitophagy induced by PINK1/Parkin.

### 3.5. Enhanced Mitophagy Ameliorates High Salt-Induced Tau Protein Hyperphosphorylation

**Figure 6:**
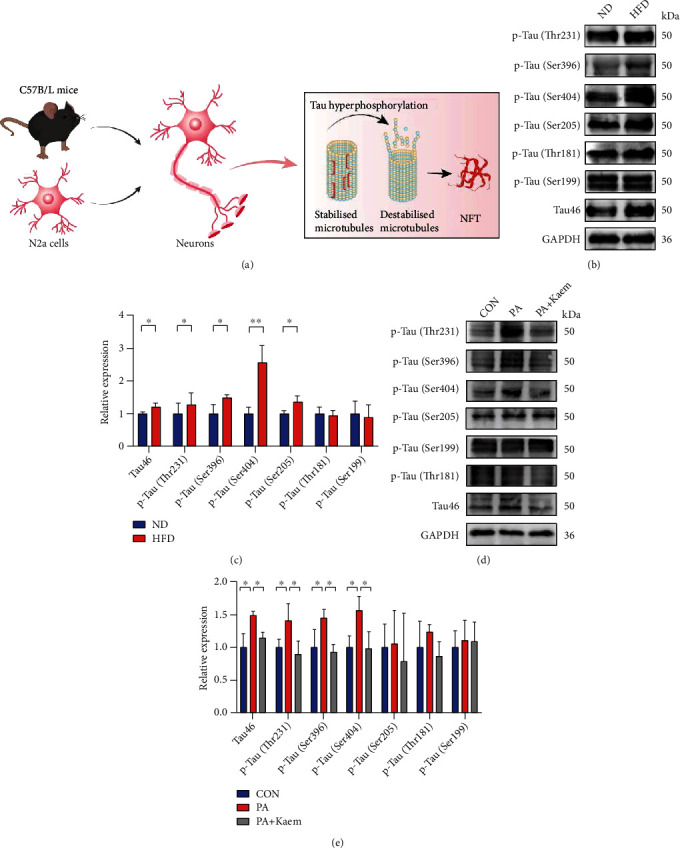
Long-term dietary fat induces tau protein hyperphosphorylation. (a) Schematic diagram of a synapse. (b, c) High-fat treatment for 6 months led to an increase in p-Tau (Thr231, Ser396, Ser205, and Ser404) in mice (*n* = 3/group, two-sample *t*-test). (d, e) The cells treated with PA (200 *μ*M) showed upregulated levels of p-Tau (Thr231, Ser396, and Ser404) compared with the CON group and high fat cells treated with Kaem (*n* = 3/group, one-way ANOVA). The relative level of proteins is normalized to GAPDH. All data are expressed as mean ± SD; ^∗^*p* < 0.05, ^∗∗^*p* < 0.01. Abbreviations: ND: normal diet; HFD: high-fat diet; CON: control; PA: palmitic acid; TNF: neurofibrillary tangles.

Western blotting was used to detect the levels of p-Tau and total Tau (Tau 46; Figure 6(a)) to detect Tau-related alterations. A significant upregulation in the protein levels of p-Tau (Thr231, Ser396, Ser205, and Ser404) was observed in the HFD group compared with the ND group. In contrast, the expression level of p-Tau (Ser199 and Thr181) was not significantly elevated after exposure to 6 months of HFD (Figures 6(b) and 6(c)). These results were also present *in vitro* (Figures 6(d) and 6(e)). Therefore, HFD leads to the phosphorylation of Tau at multiple sites. In addition, phosphorylated Tau was reduced after the addition of a mitophagy inducer *in vitro*, suggesting that hyperphosphorylation of Tau may be linked to the inhibition of mitophagy in a high-fat model of cells.

### 3.6. Dietary Fat Induces Neuronal Damage and Synapse Dysfunction, and Enhanced Mitophagy Protects Cells from PA-Induced Synaptic Damage

**Figure 7:**
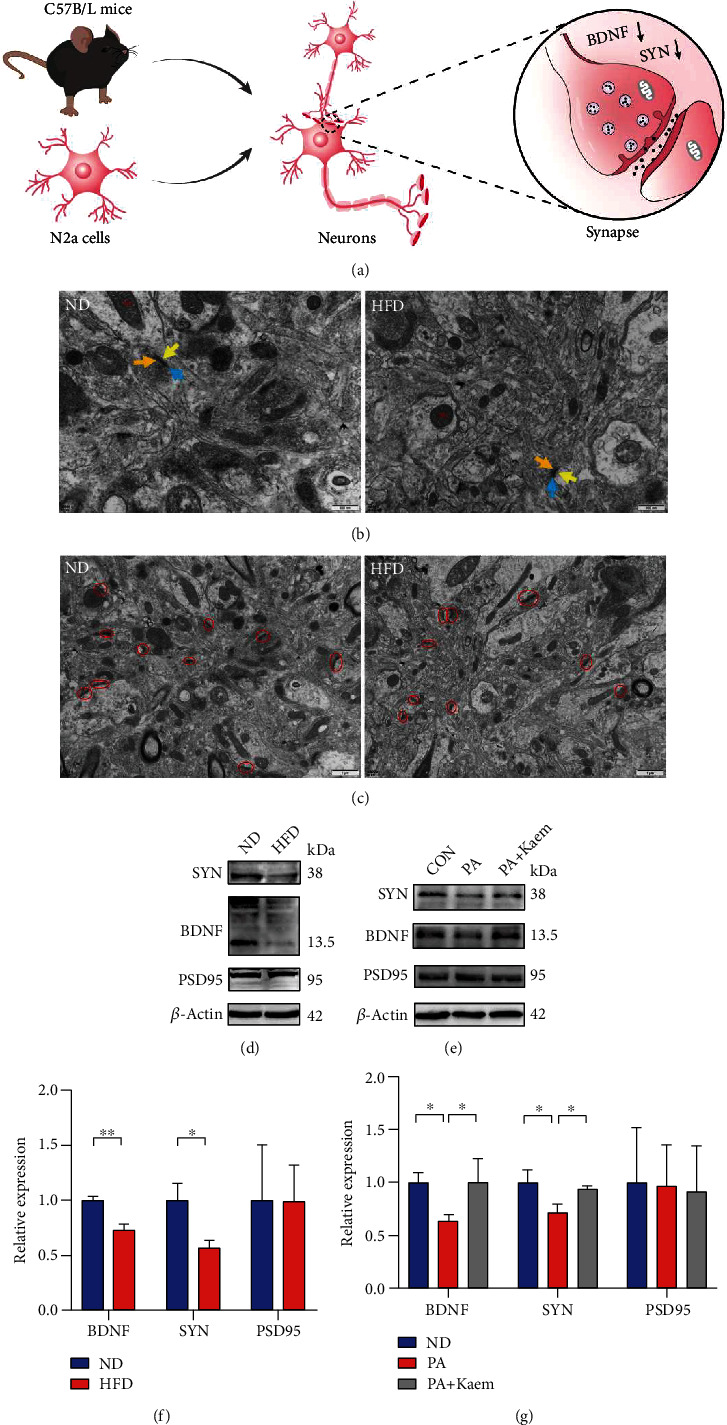
Ultrastructure synapses. (a) Diagrammatic sketch of a synapse. (b) Using transmission electron microscopy (TEM), we observed that the synaptic structure is clear and intact, and the dense material in the posterior membrane is visible. The morphological structure of the synapse is relatively intact, and no dissolution of the postsynaptic membrane-dense material is observed. Mi: mitochondria; orange arrow: presynaptic membrane; yellow arrow: postsynaptic membrane; blue arrow: synaptic cleft. (c) The synapse density (red circles) displayed no significant difference in two groups. (d, f) Western blotting detected the expression of BDNF, SYN, and PSD95 *in vivo* (*n* = 3/group, two-sample *t*-test). (e, g) The expression of BDNF, SYN, and PSD95 *in vitro* (*n* = 3/group, one-way ANOVA). All data are expressed as mean ± SD; ^∗^*p* < 0.05, ^∗∗^*p* < 0.01. Scale bar = 1 *μ*m. Abbreviations: ND: normal diet; HFD: high-fat diet; CON: control; PA: palmitic acid; BDNF: brain-derived neurotrophic factor; PSD95: postsynaptic density protein 95; SYN: synaptophysin.

The synapses of neurons in mice are illustrated in Figure 7(a). TEM showed clear and complete synaptic structures between neurons or between neurons and nonneuronal cells, including the presynaptic membrane, synaptic gap, and the postsynaptic membrane, with the presynaptic membrane containing synaptic vesicles and the postsynaptic membrane showing dense material (Figure 7(b)). In the HFD group, the synapse structure was relatively intact, the synaptic gap was clear, and the dense material of the postsynaptic membrane was not dissolved (Figure 7(b)). Although the morphology of synapses was changed in the high-fat group, the current evidence was not sufficient to demonstrate a significant decrease in the number of synapses (Figure 7(c)).

Additionally, we assessed the effects of an HFD on synapse-associated proteins and BDNF by western blotting. Figures 7(d)–7(g) show that aged mice treated with an HFD as well as N2a cells treated with PA had lower expression levels of SYN and BDNF, while the PSD95 expression did not change significantly. Combining the results of electron microscopy and western blotting, we found that dietary fat in aged C57BL/6 mice results in synaptic dysfunction and neuronal damage. Synaptic damage and reduced BDNF can contribute to spatial memory and learning capacity impairment, which is line in the results from the MWM. However, there was no significant decrease in SYN and BDNF in cells treated with a mitophagy inducer. This suggests that synaptic function may be impaired by mitophagy inhibition in response to HFD.

## 4. Discussion

The mechanisms underlying the deleterious effects of dietary fat were investigated in the brain in this study. A previous study tracked lipid droplets *in vitro* and revealed that astrocytes and neurons are susceptible to excess fatty acids [28]. In this study, we used an aged C57 mouse model that mimics continuous high lipid intake in humans. We found that the mitochondria were significantly damaged, and that the PINK/Parkin mitophagy pathway was retrained, inducing Tau protein hyperphosphorylation and synapse dysfunction during aging.

Hyperphosphorylated Tau proteins indirectly promote Tau accumulation, which segregates other cellular constituents and leads to cognitive impairment. Although it has recently been demonstrated that HFD mice showing signs of AD had an accumulation of p-Tau protein, our findings further implicate that diet habits accelerate Tau protein phosphorylation at multiple genomic loci, and the results from cellular models were consistent with these findings. Moreover, our data revealed that an HFD for 6 months induced neuronal damage and synaptic morphological changes in mice. The cause of early clinical manifestations of memory deficits may be related to synaptic disorder and loss rather than neuronal loss or accumulation of plaques and tangles [29, 30]. We found significantly reduced SYN and BDNF levels in the brains of HFD mice. BDNF has been implicated in neuronal survival, neurogenesis, differentiation, and synaptic plasticity as well as in the functional integrity of the hippocampus, as it relates to learning and memory abilities [31]. Moreover, patients with AD have been reported to exhibit deficiencies in the BDNF expression [32]. The BDNF concentration in the cerebrospinal fluid decreases with age in normal adults [33]. A recent study has shown the rejuvenating capability of young cerebrospinal fluid in old age and confirmed that this particular fluid could restore oligodendrogenesis and memory in aged mice [34]. SYN is a protein localized to presynaptic vesicles that regulates neurotransmitter release and synaptic plasticity. It is involved in synapse formation and is known to have vital effects on cognitive behavior [35]. Synaptic pathology in AD initiates at the presynaptic terminal and diffuses to postsynaptic sites [36], and Tau acts aberrantly during synaptic decline. Abnormal Tau phosphorylation compromises the transport of glutamate receptor subunits towards postsynaptic density, which subsequently contributes to synaptic loss due to decreased mitochondria-dependent ATP production [37, 38]. Our study did not reveal sufficient evidence to prove that a diet rich in fat leads to synaptic loss, but it cannot be ruled out that soluble amyloid and Tau proteins are involved in synaptic damage. Microtubule stabilizers such as paclitaxel and epothilone D exert similar effects on microtubule-associated proteins and prevent microtubule dissociation-dependent chromosomal segregation; therefore, microtubule stabilizers can be applied as anticancer therapeutic agents [39]. Anticancer drugs, including microtubule stabilizers, modulate the mitochondrial structure as well as the movement and function of cancer cells. In particular, these stabilizers can be used in the treatment of neurodegenerative diseases to combat neuronal microtubule destabilization and promote vesicle and mitochondrial transport [40].

Previous findings have demonstrated that dietary high-fat decreases the expression of autophagy-related proteins [41]. A study by Dan Shao et al. found that long-term HFD exposure inhibited Parkin-mediated mitophagy, causing the accumulation of damaged mitochondria [42]. In AD brains, the levels of PINK1 and mitophagy are decreased [18, 43]. Further, the PINK1 overexpression has been shown to alleviate cognitive dysfunction in AD mice by enhancing the removal of damaged mitochondria [44]. In the brains of AD patients and mouse models, Parkin-mediated mitophagy is induced during the early stages of the disease; nevertheless, as the disease progresses, the intracellular depletion of Parkin results in defective mitophagy and an abnormal accumulation of abnormal mitochondria in AD neurons [45]. Snapin-enhanced retrograde transport compensates for synaptic loss in the brains of AD mice by promoting axonal mitochondrial clearance and attenuating synaptic mitochondrial defects [45]. However, it has also been documented that both saturated and unsaturated fatty acids can spur autophagy through diverse mechanisms [46]. Enterocytes momentarily store dietary fat in triglyceride-containing droplets localized in the endoplasmic reticulum, and these droplets induce an immediate autophagic reaction [47], which may be an essential mechanism for preventing lipotoxicity at the cell-autonomous level [48]. Interestingly, cellular senescence can also trigger autophagy [49]. The significant variation between our current results and those in the literature may be attributed to the age, sex, and duration of HFD exposure in the mice, since the existing studies used young mice and only fed them for a short period. During lipid overload, PINK1/Parkin-mediated mitophagy continuously shields tissue from increased mitochondrial stress. However, sustained mitophagy placed an inevitable burden on the lysosomal system, possibly in the form of impaired acidification and phospholipid accumulation, which ultimately result in impaired flux of mitochondrial autophagy. Additionally, long-term administration of HFD has been known to cause insulin signaling disruption in the brain [50]. Indeed, brain insulin resistance can contribute to Tau protein hyperphosphorylation and cognitive impairment [51]. Some studies have shown that PA reduces insulin sensitivity and neuronal mitochondrial activity by increasing mitochondrial ROS production [21]. Collectively, long-term consumption of an HFD could lead to lysosomal dysfunction and consequently impair autophagy flux, which may further result in Tau phosphorylation and synaptic dysfunction and ultimately cause cognitive impairment. However, we used the mitophagy agonist only in *in vitro* experiments; thus, further *in vivo* studies are required to verify whether inhibition of mitophagy leads to cognitive impairment. In addition, our study did not include autophagy flux experiments with autophagy inhibitors. In fact, this is an exciting future area of investigation. Overall, therapies that strive to restore the autophagic flux will pave the way for better clinical outcomes in cases of HFD-induced cognitive dysfunction related to the aging process.

**Figure 8:**
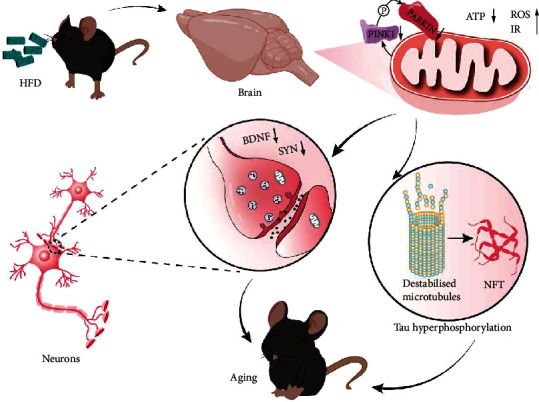
Potential mechanism of fat-induced cognition impairment. The HFD damages the mitochondria, inhibits mitophagy, and causes Tau protein phosphorylation and synaptic damage, eventually leading to cognitive dysfunction. Abbreviations: HFD: high-fat diet; BDNF: brain-derived neurotrophic factor; PSD95: postsynaptic density protein 95; SYN: synaptophysin; ATP: adenosine triphosphate; ROS: reactive oxygen species; IR: insulin resistance; NFT: neurofibrillary tangles.

In conclusion, this study proposes a previously unrecognized link between dietary habits, mitophagy, Tau pathology, and synaptic processes. Our findings present insight into the potential mechanisms of Tau protein hyperphosphorylation and synaptic damage induced by high-fat diets (Figure 8). Although the specific mechanism will need to be further elucidated, this study may lay the foundation for future research on Tau phosphorylation and synaptic dysfunction induced by high-fat diets from the perspective of energy metabolism.

## Data Availability

The data used to support the findings of this study are included within the article.
